# The assessment of body representation in adults through computer-based tasks

**DOI:** 10.3389/fpsyg.2025.1610265

**Published:** 2025-09-04

**Authors:** Simona Raimo, Mariachiara Gaita, Daniela Malangone, Erica Dolce, Lidia Ammendola, Silvia Canino, Valentina Torchia, Giada Panzino, Mariamichela Aquino, Maria Cropano, Antonella Di Vita, Maddalena Boccia, Liana Palermo

**Affiliations:** ^1^Department of Medical and Surgical Sciences, “Magna Graecia” University of Catanzaro, Catanzaro, Italy; ^2^Department of Psychology, University of Campania “Luigi Vanvitelli”, Caserta, Italy; ^3^Multiple Sclerosis Center, UOSD Second Neurology, University of Campania “Luigi Vanvitelli”, Naples, Italy; ^4^Department of Health Sciences, “Magna Graecia” University of Catanzaro, Catanzaro, Italy; ^5^UOSD Second Neurology, University of Campania Luigi Vanvitelli, Naples, Italy; ^6^Department of Human Neuroscience, Sapienza University of Rome, Rome, Italy; ^7^Department of Psychology, Sapienza University of Rome, Rome, Italy

**Keywords:** action-oriented body representation, nonaction-oriented body representation, computer based task, validation, normative data

## Abstract

**Introduction:**

This study aims to evaluate the use of computer-based body representation tasks in an adult sample, considering the role of demographic variables and providing correction indices for clinical practice.

**Method:**

Three hundred sixty-six healthy participants were assessed in person with a computer-based battery that included the Hand Laterality Task (HLT) to assess action-oriented body representation (aBR), the Frontal Body Evocation Task (FBET) to assess nonaction-oriented body representation (NaBR), and two corresponding control tasks (i.e., the Object Laterality Task and the Christmas Tree Task), to disentangle the effect of cognitive functions required to perform the tasks but independent of body representation processing. In addition to the primary cohort, 305 healthy participants performed similar body representation and control tasks in an unsupervised web-based version, and a subgroup of these (*N* = 30) underwent the assessment in both the laboratory-based and web-based versions.

**Results:**

Concerning the body representation tasks, multiple linear regression analysis revealed that age and sex significantly influenced aBR accuracy and response time (i.e., the HLT), while the NaBR accuracy and response time (i.e., the FBET) were significantly influenced only by age. A correction grid was constructed from the derived linear equation to adjust raw scores according to demographic variables, and a percentile distribution of adjusted scores was provided for each task. Correlation analyses showed significant and strong correlations between the laboratory-based and web-based versions of the tasks (*r* ≤ 0.888; *ps* < 0.001), supporting the use of these tasks for the remote assessment.

**Discussion:**

The provided normative data can be helpful for clinical and research purposes, and we discuss the potential benefits of their use.

## 1 Introduction

Our body is crucial to our sense of self and a core component of our identity (Coello, 2015). We take the perception and representation of our body for granted, as it functions mainly without conscious awareness, until something goes wrong, as in certain pathologies. Indeed, distortions and misperceptions of the body are the central features of several serious psychiatric (e.g., body dysmorphic, Phillips et al., 2008; eating disorders, Treasure et al., 2010) and neurological conditions (e.g., asomatognosia, Critchley, 1953; somatoparaphrenia, Vallar and Ronchi, 2009; personal neglect, Committeri et al., 2018; for an overview see also Palermo and Di Vita, 2023).

Neuropsychological literature suggests that the representation of the body is a multidimensional concept (Berlucchi and Aglioti, 2010; de Vignemont, 2010). Previous studies have proposed a dyadic taxonomy that distinguishes between body schema and body image (Dijkerman and de Haan, 2007; Gallagher, 2005; Paillard, 1999). The body schema, also known as action-oriented body representation (aBR; Di Vita et al., 2016), is a dynamic representation of the body, continuously updated based on sensory and motor inputs (Schwoebel and Coslett, 2005; de Vignemont, 2010). aBR plays a crucial role in regulating posture, guiding movement, and facilitating the production, recognition, and imitation of gestures. In contrast, body image, also referred to as nonaction-oriented body representation (NaBR) (Di Vita et al., 2016), encompasses all perceptual, conceptual, and emotional representations of the body that are not directly linked to action. NaBR is responsible for localizing body parts, defining body boundaries in relation to the external environment, and determining body proportions, shape, and weight. Additionally, NaBR plays a critical role in tasks requiring the visual recognition of one’s body and is instrumental in determining the felt location of sensations, allowing them to be attributed to specific body parts via structural body representation (Pitron and de Vignemont, 2017). Due to the complexity and heterogeneity of body image, a triadic taxonomy has been suggested, subdividing body image into two distinct representations: (i) a visuo-spatial map of the body (also called body structural representation), which is a topographic representation primarily derived from visual information about body part boundaries and proximity relationships; and (ii) a lexical–semantic representation of the body, encompassing body part names, functions, and relationships with external objects (Schwoebel and Coslett, 2005; Sirigu et al., 1991).

Although various taxonomies and models have been suggested, the distinction between aBR and NaBR remains the most frequently used for cognitive and neuropsychological investigations of body representation (Di Vita et al., 2016; Palermo et al., 2018; Pitron and de Vignemont, 2017; Sattin et al., 2023).

Assessing body representation is inherently challenging due to the multiple facets involved, each requiring specific measurement tools. In clinical practice, body representation disorders are often assessed with instruments based on the clinical features of the disorders more than on body representation taxonomies and cognitive architecture (for such an argument, see Palermo and Di Vita, 2023; Serrada et al., 2023); often these instruments tax more body representations. For example, one of the most commonly used tasks to assess body representation is the Pointing to One’s Body Part task, in which participants are asked to indicate a body part that has been touched, named, or visually presented in an image (Anema et al., 2009; Head and Holmes, 1911; Paillard, 1999; Semenza and Goodglass, 1985). However, as suggested by de Vignemont (2010), it is not clear what specific body representation this task is supposed to assess. Indeed, on the one hand, this task has been considered as a measure of aBR, as suggested by the poor performance observed in patients with deafferentation, but not in those with numbsense (Dijkerman and de Haan, 2007; Gallagher and Cole, 1995; Paillard, 1999); on the other hand, it has also been considered as a measure of NaBR, as suggested by the difficulties shown in this task by patients with autotopagnosia, but not by those with apraxia (Schwoebel and Coslett, 2005). “Exclusive (i.e., specific to one kind of body representation only)” (de Vignemont, 2010, p. 7) instruments are thus necessary to identify the body representation difficulty underlying a clinical disorder. In this vein, some tasks have been developed to evaluate specific body representations, with distinct instruments designed to assess aBR or NaBR. For example, in experimental settings, a common way to evaluate the aBR is by using motor imagery tasks, in which individuals mentally visualize their body performing specific movements (Schwoebel et al., 2002; Schwoebel and Coslett, 2005). Indeed, substantial evidence suggests that the mental simulation of body movements is guided by the body schema, much like actual physical actions (Parsons, 1994; Parsons and Fox, 1998; Schwoebel et al., 2001). Among motor imagery tasks, the Hand Laterality Task (HLT) has been extensively used to examine the body schema. In this task, participants judge the laterality of a visually presented hand by mentally rotating their own imagined hand (Parsons, 1987). The HLT is particularly helpful for assessing the ability to organize actions within spatial contexts, as it involves body schema in planning and executing movements. The HLT has proven valuable in assessing aBR deficits across various clinical populations, including adults with brain damage (Boccia et al., 2020; Raimo et al., 2022; Razmus, 2017; Schwoebel and Coslett, 2005), children with cerebral palsy (Fontes et al., 2017; Di Vita et al., 2020), individuals with chronic arm pain (Schwoebel et al., 2001), and patients with upper-limb amputations (Nico et al., 2004). A key strength of the HLT is the possibility to include control tasks, which require participants to mentally rotate non-body-related stimuli (e.g., letters, abstract shapes, or flowers with asymmetrically positioned leaves; Conson et al., 2020; Raimo et al., 2022; Schmid and Coppieters, 2012). This allows researchers to determine whether performance deficits stem from impaired body representation or other cognitive processes necessary for task completion. The HLT, therefore, provides a robust measure of the body representation involved in executing movements and actions and the underlying neural mechanisms. Various tasks have also been developed to assess NaBR (Schwoebel and Coslett, 2005; Razmus, 2017; Palermo and Di Vita, 2023), which is a body representation primarily based on visual experience. For example, questionnaires are commonly used to assess body image-related perceptions, such as body satisfaction (Body Image Scale; Thompson et al., 1999) or subjective feelings about one’s body (Body Image Disturbance Questionnaire; Cash and Smolak, 2011). Additionally, tasks measuring the visuo-spatial body map or structural body representation have been developed, such as the Matching Body Parts by Location Task (see Fontes et al., 2017; Schwoebel and Coslett, 2005; Razmus, 2017), in which participants are shown a body part and asked to choose, from different options, the one that physically continues from it, or tasks that evaluate the metric characteristics of body parts (for an overview see Sorrentino et al., 2021). Among these last ones, the Frontal Body-Evocation Task (FBET) of the Body Representation Test (Daurat-Hmeljiak et al., 1978) is a task with standardized administration and scoring procedures that has proved to be useful to assess NaBR alterations in different kinds of clinical populations, such as adult patients with unilateral brain damage (e.g., Di Vita et al., 2017, 2019; Guariglia and Antonucci, 1992; Guariglia et al., 2002; Marangolo et al., 2003), patients with achondroplasia before and after surgical elongation of lower limbs (Di Russo et al., 2006) and patients with lower limb amputation (Palermo et al., 2014). In the standard paper and pencil version, participants, after viewing the picture of a human body, have to accurately locate nine different body parts (legs, hands, and so on) on a board depicting only the head; for each part correctly located, one point is assigned. A computerized version of this task has also been developed, including a similar control task without body stimuli (i.e., the Christmas Tree Task; see Raimo et al., 2021a,^2022^). This version uses a more fine-grained accuracy measure since the accuracy of the answer is recorded for each body part in terms of mm deviations from the correct location of the body part. This version also allows for a more precise assessment of changes in NaBR over time. The strength of the FBET lies in its exclusive focus on the static representation of the body, intentionally excluding motor aspects. This specificity enables the identification of alterations in the ability to mentally evoke and organize body image. Furthermore, the possibility of including a control task, such as the Christmas Tree Task—which does not involve bodily stimuli—enhances the validity of the assessment by isolating specific NaBR deficits and minimizing the risk of confounding factors.

Although several of these tasks have been employed to evaluate body representations in both clinical (Schwoebel and Coslett, 2005; Razmus, 2017; Boccia et al., 2020; Raimo et al., 2022, 2024) and non-clinical populations (Canino et al., 2022; Raimo et al., 2021a,b,c), no normative study has yet been conducted incorporating tasks that assess both NaBR and aBR alongside control tasks. Indeed, to the best of our knowledge, a standardized version of the HLT has been developed for in-person and online assessment (Moreno-Verdú et al., 2025), but this study did not consider demographic variables or the inclusion of a specific control task.

Following Bonato et al. (2012), using control tasks is particularly relevant to prevent a potential fallacy, namely, erroneously attributing impaired performance to a specific pathology. Indeed, for example, the poor performance of a patient with right brain damage as compared with healthy controls in a task assessing the visuo-spatial body map, such as the FBET, could be due to a more general deficit in visuo-spatial processing and not to the pathology in body representation itself. This risk can be reduced by assessing a patient or a group of patients also using a “control task similar to the experimental one in terms of setting (stimuli presentation, response modality) and task difficulty, but requiring cognitive processes differing from those that are the object of the study” (Bonato et al., 2012). Consistently, it has been suggested that the assessment of aBR and NaBR by means of specifically developed tasks, including control tasks, may help clinicians to identify better body representation alterations in patients with central or peripheral nervous system disorders (Raimo et al., 2022), thus improving the overall treatment options and quality of life. Also, the relevance of an unsupervised web-based assessment is becoming increasingly clear after the COVID-19 pandemic, since it can offer advantages such as better accessibility and inclusivity for vulnerable and isolated individuals and saving time and resources. However, issues remain regarding the validity of such tools, and many authors underline that it is crucial to invest in validating and providing normative data for web-based measures (see Belleville et al., 2023).

Therefore, based on these considerations and on a recent review that underlines the lack of robust assessment tools (Serrada et al., 2023), the present study had two main objectives. First, it aimed to validate and provide normative data for two specific computerized tasks designed to assess both aBR and NaBR (i.e., HLT and FBET), also considering paired control tasks. Second, the study aimed to compare the performance of these tasks across two administration modalities, laboratory-based and web-based, in order to assess the feasibility and validity of remote, unsupervised assessment. We hypothesized that (i) performance on the body representation tasks would be significantly influenced by demographic variables, including age, sex, and education, consistent with findings in other cognitive domains, and (ii) the web-based version of the tasks would show strong convergent validity with the laboratory-based version, supporting the use in remote assessment settings.

## 2 Materials and methods

### 2.1 Participants

An a priori power analysis was conducted using G*Power 3.1 to determine the minimum required sample size for the regression analyses. Based on a model with three independent variables (age, education, and sex), an alpha level of 0.05, a statistical power of 0.80, and a small-to-moderate expected effect size (*f*^2^ = 0.04), the analysis indicated that at least 277 participants were needed.

Three hundred sixty-six healthy individuals from different Italian districts (most in the South of Italy) took part in the study (178 male participants and 188 female participants), covering a broad age range (18–80 years) and representing different levels of formal education (from primary school to university). They were recruited through public advertisements posted online (e.g., social media, research volunteer platforms) and local community centers. Screening was conducted through a brief structured clinical interview and two cognitive screening tools: the Montreal Cognitive Assessment (MoCA) and the Raven’s Colored Progressive Matrices (RCPM). Participants were excluded if they reported any history of psychiatric or neurological disorders, according to the Diagnostic and Statistical Manual of Mental Health Disorders, 5th Edition (DSM-5; American Psychiatric Association [APA], 2013); had uncorrected visual or auditory impairments; or scored below clinical cutoffs on the Montreal Cognitive Assessment (MoCA) (Nasreddine et al., 2005) according to the Italian normative data (Santangelo et al., 2015), and on the Raven’s Colored Progressive Matrices (RCPM) (Raven, 1947) according to the Italian normative data (Spinnler and Tognoni, 1987), to exclude the presence of general cognitive impairment and deficit in abstract reasoning.

The whole sample had a mean age of 49.12 years (±17.73) and a mean formal education of 12.86 years (±3.69). The distribution of the sample for age, education, and sex is reported in Table 1.

**TABLE 1:** Demographic distribution of the entire sample (*N* = 366) underwent body representation and control tasks in the lab-based version.

Demographic variables	Age (years)												
	18–30		31–40		41–50		51–60		61–70		71–80		Total
Education (years)	M	F	M	F	M	F	M	F	M	F	M	F	
0–8	−	2	3	3	5	8	3	9	2	4	11	18	68
9–13	18	27	14	14	19	19	19	13	20	18	14	8	203
>13	10	4	15	16	7	7	7	7	7	10	4	1	95
Total	28	33	32	33	31	34	29	29	29	32	29	27	366

The table reports the number of male (M) and female (F) participants across six age bands (18–30, 31–40, 41–50, 51–60, 61–70, 71–80) and three education levels (0–8, 9–13, > 13 years of education). Each cell indicates the count of participants by age, sex, and education. The final column and row provide totals.

In addition to this primary cohort, we extended our evaluation to another sample of healthy participants (*N* = 305; for detailed demographics, see Supplementary Material 1) who underwent similar body representation and control tasks in a web-based version. A subgroup of these participants (*N* = 30), with a mean age of 46.19 years (±16.22) and mean formal education of 14.19 years (±2.23), underwent the assessment in both laboratory-based and web-based settings, in randomized order. This secondary assessment was conducted 6 weeks following the initial evaluation.

The study was performed in conformity with the local Ethics Committee requirements (Calabria Region Ethical Committee, Catanzaro, Italy) and the criteria set down in the 1964 Declaration of Helsinki.

### 2.2 Procedure

All participants were submitted to a computerized battery that included two body representation tasks: the HLT, and the FBET to evaluate the aBR and the NaBR, respectively. Moreover, two corresponding control tasks (the Object Laterality Task, OLT; and the Christmas Tree Task, CTT) were also administered. The control tasks were similar to the body representation tasks in terms of their presentation and response features but did not involve body processing. Tasks are briefly described below, and an accurate description of the tasks and procedures can be found in previous studies from our research group (for the laboratory-based version of the tasks, see Raimo et al., 2021a,^2022^; for the web-based version of the tasks, see Canino et al., 2022, Raimo et al., 2023).

In the lab-based assessment, participants were evaluated in person by an experimenter using a laptop (13.3″ display) with a touchscreen monitor; the laptop was placed on a desk in front of the participants. They were invited to respond immediately after the presentation of the stimuli, even if they had no time limit.

In the web-based setting, participants completed the tasks unsupervised on the Testable platform using their computers (desktop or laptop) with either a mouse or a touchpad as an input device. Before beginning the tasks, participants completed a visual calibration step: they were instructed to place a standard physical object (either their national health card or electronic ID card, both conforming to ISO/IEC 7810 ID-1 format, 85.60 × 53.98 mm) against the screen, and to adjust an on-screen reference line until it matched the real-world card size. This ensured that stimulus dimensions were standardized across screen resolutions and sizes.

Although the web-based setting involved unsupervised administration, several strategies were adopted to enhance standardization and data reliability. All participants received detailed instructions encouraging them to complete the task in a quiet setting using a desktop or laptop device. A visual calibration step ensured consistency in stimulus presentation across screens. Additionally, task design was robust to variations in input method (mouse or touchpad), and performance data were screened for anomalies.

All participants (100%) completed every task.

The average duration of the testing (i.e., HLT, FBET, OLT and CTT) was approximately 15 min both in the web-based setting, and in the laboratory setting.

#### 2.2.1 In-person assessment of body representations in the laboratory setting

##### 2.2.1.1 Assessment of the aBR (body schema)

The aBR was assessed using the HLT (adapted and simplified from Parsons, 1987; see Raimo et al., 2021a,^2022^). In this task, the participants were asked to judge the laterality of a single hand (20 stimuli, 10 left hands and 10 right hands) that could be presented in five different angles of rotation (0, 45, 90, 270, 315 degrees) on a computer screen. Specifically, participants had to decide whether the target stimulus was a left or a right hand by mentally rotating it and indicating the answer by tapping a left or right hand shown at the bottom of the screen (not rotated). In the correspondent control task, the OLT (see Raimo et al., 2021a,^2022^), involving a mental rotation task of non-body-related stimuli, the participants were asked to judge the laterality of a flower with a leaf positioned at the right or at the left base of the stem (20 stimuli, 10 flowers with a leaf positioned at the left and 10 flowers with a leaf positioned at the right) that could be presented in five different angles of rotation (0, 45, 90, 270, 315 degrees) on a computer screen. Specifically, participants had to decide whether the target flower corresponded to the one with the leaf positioned at the left or the right base of the stem and answer by tapping on one of the two response options at the bottom of the screen.

**FIGURE 1:**
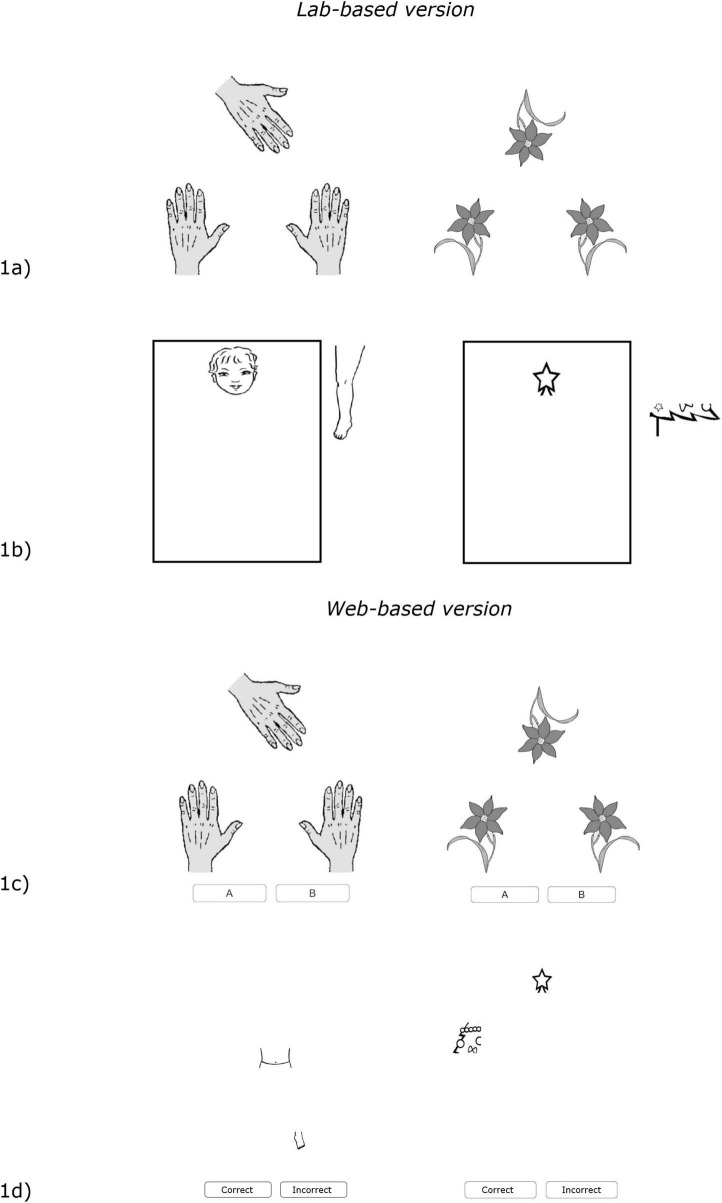
Examples of items for the tasks involving body (right) and non-body (left) processing in lab-based and web-based versions. The upper panel shows the lab-based version of the tasks: **(a)** an item of the task assessing the body schema (Hand Laterality Task) is shown on the left panel and an item of the control task (Object Laterality Task) is shown on the right panel; **(b)** the task assessing the body structural representation (Frontal Body Evocation Task) is shown on the left panel and the control task (Christmas Three Task) on the right panel. The lower panel shows the web-based version of the tasks: **(c)** an item of the web task assessing the body schema (Hand Laterality Task) is shown on the left panel and an item of the control task (Object Laterality Task) is shown on the right panel; **(d)** the web task assessing the body structural representation (Frontal Body Evocation Task) is shown on the left panel and the control task (Christmas Three Task) on the right panel.

In both tasks, individual accuracy corresponded to the sum of correct responses; individual scores ranged from 0 to 20, with higher scores indicating better performance. In both tasks, participants were given two practice items to ensure they understood the instructions, followed by the 20 test items. The order of presentation of these tasks was counterbalanced across participants, and response times were also recorded (see Figure 1a for an example of the tasks).

##### 2.2.1.2 Assessment of the NaBR (body structural representation)

The NaBR was assessed using the computerized version of the FBET (modified from the paper and pencil version by Daurat-Hmeljiak et al., 1978; see Raimo et al., 2021a,^2022^). In this version, the participants observed a picture of a human body for 10 s, and then they were asked to re-locate a specific body part (i.e., the left or right leg, hand, arm, part of the chest, or the neck) by dragging it on a touchscreen where only the head was shown as a reference point. Participants were presented with one specific body part at a time, and before presenting a new body part, the computer recorded the position of the located body part.

In the paired control task, the Christmas Tree Task, involving the visuospatial processing of non-body related stimuli (see Raimo et al., 2021a,^2022^), the participants observed a picture of a Christmas tree for 10 s; they were then asked to re-locate a specific part of the tree (left or right lower branches, middle branches, lower branches with trunks, parts of the jar, or the top) by dragging it with a finger on a touchscreen where only the star tree topper serves as a reference point on the touchscreen during this task. Participants were presented with one specific Christmas tree part at a time, and, before presenting a new Christmas tree part, the computer recorded the position of the located part.

In both tasks, accuracy was measured as the deviation (in millimeters, mm) from the correct location (a smaller deviation in mm indicated better performance). The order of presentation of these tasks was counterbalanced across participants, and response times were also recorded (see Figure 1b for an example of the tasks).

#### 2.2.2 Unsupervised assessment of body representations in the web-based setting

##### 2.2.2.1 Assessment of the aBR (body schema)

In the web-based version of the HLT (Canino et al., 2022; Raimo et al., 2023), as for the lab-based assessment, the participants were asked to make a decision on the laterality of a single hand presented at varying degrees of angular rotation (0°, 45°, 90°, 135°, 180°, 225°, 270°, and 315°). Specifically, participants had to decide whether the target stimulus was a left or a right hand by mentally rotating it and indicating the answer by selecting one of two response buttons (A and B) next to a left and right hand shown at the bottom of the screen. The task included 48 trials (24 left hand and 24 right hand).

Similarly, in the web-based version of the OLT (Canino et al., 2022; Raimo et al., 2023), the control task that includes the mental rotation of non-body stimuli, the participants were asked to make a decision on the laterality of a flower with a leaf positioned at the right or left base of the stem, presented at varying degrees of angular rotation (0°, 45°, 90°, 135°, 180°, 225°, 270°, and 315°). Specifically, participants had to decide whether the target flower corresponded to the one with the leaf positioned at the left or the right base of the stem and answer by selecting one of the two response options at the bottom of the screen marked with the letters A and B.

The task included 48 trials (24 flowers with a leaf positioned at the left of the stem and 24 flowers with a leaf positioned at the right of the stem).

In both tasks, participants were given four practice items with feedback to ensure they understood the instructions, followed by the 48 test items. One point was assigned for each correct response, with higher scores indicating better performance (maximum score: 48). The task presentation order was counterbalanced across participants, and response times were also recorded (see Figure 1c for an example of the task items).

##### 2.2.2.2 Assessment of the NaBR (body structural representation)

In the web-based version of the FBET (Canino et al., 2022; Raimo et al., 2023), as for the laboratory in-person assessment, the participants were shown a drawing of a body for 10 s. Then, they were asked to indicate whether a specific body part was correctly or incorrectly located relative to the head or the torso used as a reference point. Each body part (i.e., the right or left hand, arm, leg and foot) was presented six times in different positions: correct, for a total of 16 stimuli (8 with the torso as the point of reference, and 8 for the head as the point of reference); incorrect with a minimal deviation from the right location, for a total of 16 stimuli (8 with the torso as point of reference, and 8 for the head as point of reference); incorrect with a significant deviation from the right location, for a total of 16 stimuli (8 with the torso as point of reference, and 8 for the head as point of reference).

In the web-based version of the CTT (Canino et al., 2022; Raimo et al., 2023), as for the laboratory in-person assessment, the participants observed a picture of a Christmas tree for 10 s. Then, they were asked to indicate whether a specific part of the tree was correctly or incorrectly located relative to the star tree topper or the pot used as a reference point. Each part of the tree (i.e., the lower branches on the left or right, middle branches on the left or right, upper branches on the left or right and middle branch) was presented six times in different positions: correct, for a total of 16 stimuli (8 with the star tree topper as reference, and 8 with the jar as reference); incorrect with a minimal deviation from the right location, for a total of 16 stimuli (8 with the star tree topper as reference, and 8 with the jar as reference); incorrect with a significant deviation from the right location, for a total of 16 stimuli (8 with the star tree topper as reference, and 8 with the jar as reference).

In both tasks, participants were given four practice items with feedback to ensure they understood the instructions, followed by the 48 test items. One point was assigned for each correct response, with higher scores indicating better performance (maximum score: 48). The task presentation order was counterbalanced across participants, and response times were also recorded (see Figure 1d for an example of the task items). Some procedural modifications were implemented in the web-based versions of the tasks to ensure usability and participant autonomy in unsupervised testing environments. In particular, the drag-and-drop response format used in the FBET and CTT was replaced with a binary yes/no response. This design choice followed extensive pilot testing and was motivated by the need to reduce participant dropout, usability issues, and technical errors that could arise with drag-and-drop interactions, especially on non-touchscreen devices.

Importantly, while the response modality changed, the underlying cognitive construct and decision process remained the same.

### 2.3 Statistical analysis

Statistical analysis was performed following the procedure adopted by Capitani (1997). Multiple regression analyses were conducted to assess the relative influence of demographic variables such as sex, age, and educational level on the participants’ body representation and control task accuracy measures and response times (i.e., the HLT and the OLT; the FBET and the CTT).

The effects of age and educational level (expressed as years of schooling) were explored after several transformations (e.g., logarithmic, quadratic). Sex, age and education were entered into a multiple linear regression analysis to partial out their possible overlapping effect. *R*^2^ values were interpreted according to Cohen (1988) criteria as follows: values below 0.13 indicate a small effect size, values between 0.13 and 0.26 indicate a medium effect size, and values above 0.26 indicate a large effect size.

The results of the multiple regression analyses were entered into a regression equation to calculate a correction factor for each subject of the sample. Adjusted scores were obtained by adding or subtracting the contribution of concomitant variables from the original scores. After correcting all the raw scores, the adjusted scores were ranked from the worst to the best, and a non-parametric procedure (Ackermann, 1985), with a set of confidence at 95%, was used to estimate unidirectional limits of tolerance that discriminate a score as normal or abnormal according to falling within the highest 95% or within the lowest 5% of the normal population (Capitani, 1997; Cohen, 1988). Specifically, a 95% confidence level was used to estimate unidirectional tolerance limits that distinguish a score as normal or abnormal based on whether it falls within the top 95% or the bottom 5% of the normal population. A correction grid was constructed to facilitate the adjustment of the raw scores of newly tested participants according to demographic variables. This grid includes sex, education levels, in accordance with the Italian school system, and various combinations of age (in 10-year increments). The use of 10-year age bands is consistent with previous normative studies (Bezdicek et al., 2012; O’Connell et al., 2022), and was chosen to preserve both statistical reliability, by maintaining adequate subgroup sizes, and clinical usability, by keeping the stratification easily applicable in clinical practice.

To explore the degree of convergence between the laboratory-based and web-based assessment, convergent validity was calculated using Spearman’s correlation analysis only in the subgroup of healthy participants that underwent both the computer-based and web-based versions of the tasks. The effect size for the correlation coefficient was defined by the following criteria: *r_*s*_* < 0.3 weak; *r*_*s*_ = 0.3–0.5 moderate; *r*_*s*_ > 0.5 strong (Cohen, 1988).

## 3 Results

### 3.1 Performance accuracy

Table 2 shows the descriptive statistics for the demographic data, aBR, NaBR, and control task scores, while Table 3 shows aBR, NaBR, and control task scores divided by sex, age and education. Both tables summarize data from the sample assessed in the laboratory setting.

**TABLE 2:** Means and standard deviations of demographic variables and body representation tasks for the entire sample (*N* = 366) in the lab-based setting.

Demographic variables/Task		Mean (SD)	Range (Min-max)
Age (years)		49.12 ± 17.73	18−84
Education (years)		12.86 ± 3.69	3−25
**Body representation**			
***NaBR (body structural representation)***			
Frontal Body Evocation Task	*Total mm of deviation from the correct location*	92.04 ± 50.04	26–380
	*Total response time (sec.)*	50.35 ± 31.10	15–227
Christmas Tree Task (control task)	*Total mm of deviation from the correct location*	137.15 ± 55.85	47–451
	*Total response time (sec.)*	56.15 ± 34.42	15–212
***aBR (body schema)***			
Hand Laterality Task	*Correct responses*	18.55 ± 2.83	2–20
	*Total response time (sec.)*	54.24 ± 41.37	15–286
Object Laterality Task (control task)	*Correct responses*	18.83 ± 2.85	0–20
	*Total response time (sec.)*	51.33 ± 33.61	0.79–214

The table includes descriptive statistics (mean ± standard deviation and min–max range) for demographic variables and performance on body representation tasks in the lab-based version, for the full sample of healthy controls (*N* = 366). Performance in the Frontal Body Evocation Task and Christmas Tree Task is measured in millimeters of deviation from correct location, with lower values indicating higher accuracy, and total response time in seconds. Performance in the Hand Laterality and Object Laterality Tasks is measured by the number of correct responses, with higher values indicating better performance, and total response time in seconds. HC, Healthy Controls; NaBR, Nonaction-Oriented Body Representation; Abr, Action-Oriented Body Representation.

**TABLE 3:** Descriptive statistics of the accuracy measures for body representation and control tasks (lab-based version) by age, education and sex for the entire sample (*N* = 366).

Education	Task							
	Age 18–30		Age 18–30		Age 18–30		Age 18–30	
	Frontal Body Evocation Task		Christmas Tree Task		Hand Laterality Task		Object Laterality Task	
	**M**	**F**	**M**	**F**	**M**	**F**	**M**	**F**
0–8	–	38.14 ± 1.84	–	84.47 ± 20.91	–	19.50 ± 0.70	–	20.00 ± 0.01
9–13	74.28 ± 36.11	74.46 ± 37.77	106.89 ± 38.19	122.22 ± 48.09	19.50 ± 1.04	19.41 ± 1.24	19.44 ± 1.14	18.93 ± 3.03
> 13	67.33 ± 33.05	59.14 ± 18.53	121.23 ± 58.60	98.40 ± 23.30	19.70 ± 0.48	19.50 ± 0.57	18.70 ± 3.09	20.00 ± 0.01
**Education**	**Age 31–40**		**Age 31–40**		**Age 31–40**		**Age 31–40**	
	**Frontal Body Evocation Task**		**Christmas Tree Task**		**Hand Laterality Task**		**Object Laterality Task**	
	**M**	**F**	**M**	**F**	**M**	**F**	**M**	**F**
0–8	64.98 ± 32.65	75.79 ± 18.50	126.89 ± 12.64	138.28 ± 66.59	18.67 ± 2.30	16.33 ± 6.35	18.00 ± 2.00	19.33 ± 0.57
9–13	91.23 ± 83.84	73.66 ± 31.90	121.50 ± 47.36	126.27 ± 29.82	19.21 ± 1.18	18.86 ± 1.65	19.36 ± 1.64	18.86 ± 4.27
> 13	75.98 ± 32.33	71.70 ± 26.32	119.17 ± 34.31	110.59 ± 33.84	19.07 ± 1.48	19.50 ± 0.63	19.33 ± 1.83	19.50 ± 0.96
**Education**	**Age 41–50**		**Age 41–50**		**Age 41–50**		**Age 41–50**	
	**Frontal Body Evocation Task**		**Christmas Tree Task**		**Hand Laterality Task**		**Object Laterality Task**	
	**M**	**F**	**M**	**F**	**M**	**F**	**M**	**F**
0–8	81.52 ± 22.09	82.07 ± 42.87	139.72 ± 42.09	145.94 ± 36.15	16.80 ± 5.54	18.38 ± 1.76	18.00 ± 3.46	19.75 ± 0.46
9–13	82.72 ± 35.63	67.46 ± 41.48	141.55 ± 51.06	117.53 ± 43.36	19.11 ± 1.66	18.95 ± 2.04	18.68 ± 2.68	18.84 ± 2.85
> 13	69.41 ± 21.67	67.51 ± 19.58	123.08 ± 48.86	99.13 ± 31.88	19.71 ± 0.48	19.57 ± 0.53	19.86 ± 0.37	19.43 ± 0.78
**Education**	**Age 51–60**		**Age 51–60**		**Age 51–60**		**Age 51–60**	
	**Frontal Body Evocation Task**		**Christmas Tree Task**		**Hand Laterality Task**		**Object Laterality Task**	
	**M**	**F**	**M**	**F**	**M**	**F**	**M**	**F**
0–8	78.68 ± 14.34	76.51 ± 37.93	245.41 ± 164.68	136.24 ± 61.26	17.33 ± 4.61	16.44 ± 4.55	18.67 ± 2.30	18.00 ± 3.74
9–13	95.61 ± 34.57	87.21 ± 28.91	159.29 ± 65.88	143.41 ± 43.85	18.42 ± 3.67	18.31 ± 2.39	19.37 ± 1.42	19.23 ± 1.36
> 13	102.92 ± 50.32	83.64 ± 31.66	144.51 ± 47.79	121.40 ± 43.26	18.86 ± 1.67	17.29 ± 3.68	19.43 ± 0.78	17.71 ± 3.98
**Education**	**Age 61–70**		**Age 61–70**		**Age 61–70**		**Age 61–70**	
	**Frontal Body Evocation Task**		**Christmas Tree Task**		**Hand Laterality Task**		**Object Laterality Task**	
	**M**	**F**	**M**	**F**	**M**	**F**	**M**	**F**
0–8	111.68 ± 31.30	102.43 ± 31.08	104.03 ± 40.36	242.81 ± 150.28	16.50 ± 4.95	13.25 ± 8.61	14.00 ± 8.48	18.00 ± 1.41
9–13	99.55 ± 30.67	119.65 ± 77.95	135.72 ± 51.52	158.96 ± 56.14	19.50 ± 0.76	17.89 ± 2.92	19.75 ± 0.55	16.78 ± 6.22
> 13	111.99 ± 83.68	96.31 ± 46.51	167.17 ± 73.90	128.64 ± 34.89	19.86 ± 0.37	18.40 ± 3.68	19.00 ± 1.41	18.40 ± 3.68
**Education**	**Age 71–80**		**Age 71–80**		**Age 71–80**		**Age 71–80**	
	**Frontal Body Evocation Task**		**Christmas Tree Task**		**Hand Laterality Task**		**Object Laterality Task**	
	**M**	**F**	**M**	**F**	**M**	**F**	**M**	**F**
0–8	128.76 ± 61.66	161.09 ± 60.81	155.20 ± 60.23	187.41 ± 68.92	17.36 ± 4.08	15.72 ± 5.06	16.45 ± 4.90	18.50 ± 3.18
9–13	136.01 ± 35.73	116.47 ± 65.59	160.32 ± 29.96	129.21 ± 29.89	19.36 ± 1.15	16.38 ± 3.88	19.71 ± 0.61	17.87 ± 3.44
> 13	142.74 ± 41.23	–	177.51 ± 35.79	–	19.50 ± 0.57	–	18.75 ± 1.50	–

Values are presented as mean ± standard deviation. Performance in the Frontal Body Evocation Task and Christmas Tree Task is measured in millimeters of deviation from correct location, with lower values indicating higher accuracy. Performance in the Hand Laterality and Object Laterality Tasks is measured by the number of correct responses, with higher values indicating better performance. For the number of male (M) and female (F) participants across the six age bands and the three education levels (see Table 1).

A regression model was constructed for the total score on the HLT, which revealed that age, education and sex were significant in predicting the HLT score. No demographic variables (i.e., age, education, and sex) predicted OLT performance.

Age significantly influenced the FBET performance, while education and sex did not significantly affect it. Concerning the CTT, age and education significantly predicted performance, while sex did not.

Overall, these results suggest that age is a significant predictor across different tasks, while the effects of education and sex may vary depending on the specific task (see Table 4).

**TABLE 4:** Multiple linear regression and predicting coefficients of the age, education, and sex for the body representation and control tasks (lab-based version) for the entire sample (*n* = 366).

Task		*R*	*R* ^2^	*F*	*p*	Predictor	Percentage of variance (%)	Beta (unstandardised coefficients)	*p*
***NaBR (body structural representation)***									
Frontal Body Evocation Task	*Total mm of deviation from the correct location*	0.427	0.182	26.964	** < 0.001**	Sex		−3.469	0.469
						Age	15.3%	12.030	** < 0.001**
						Education		−4.245	0.257
	*Total response time (sec.)*	0.562	0.316	55.774	** < 0.001**	Sex		0.134	0.961
						Age	27%	9.916	** < 0.001**
						Education		−3.173	0.136
Christmas Tree Task (control task)	*Total mm of deviation from the correct location*	0.344	0.119	16.226	** < 0.001**	Sex		−4.303	0.438
						Age	7.2% 15%	9.143	** < 0.001**
						Education		−11.904	**0.006**
	*Total response time (sec.)*	0.595	0.354	66.066	** < 0.001**	Sex		−3.077	0.294
						Age	31.7%	11.907	** < 0.001**
						Education		−1.387	0.545
***aBR (body schema)***									
Hand Laterality Task	*Correct responses*	0.344	0.112	15.232	** < 0.001**	Sex	1.7%	−0.732	**0.010**
						Age	73.8 4.1%	−0.284	**0.001**
						Education		0.882	** < 0.001**
	*Total response time (sec.)*	0.462	0.213	32.789	** < 0.001**	Sex	1.3%	9.537	**0.014**
						Age	17.3%	10.565	** < 0.001**
						Education		−3.056	0.314
Object Laterality Task (control task)	*Correct responses*	0.151	0.023	2.812	** < 0.001**	Sex		−0.321	0.283
						Age		−0.167	0.068
						Education		0.312	0.182
	*Total response time (sec.)*	0.387	0.150	21.331	** < 0.001**	Sex		−4.469	0.212
						Age	6.3%	7.943	** < 0.001**
						Education		−2.488	0.373

For each outcome, the table reports the multiple correlation coefficient (*R*), the proportion of explained variance (*R*^2^), the F-statistic, and associated *p*-value for the full model. Unstandardised regression coefficients (Beta), individual *p*-values, and percentage of variance explained by each significant predictor are also shown where applicable. Performance measures include total response times (in seconds), deviation from correct location (in millimeters) for the Frontal Body Evocation Task and Christmas Tree Task, and number of correct responses for the Hand Laterality and Object Laterality Tasks. Statistically significant *p*-values (*p* < 0.05) are reported in bold.

In order to allow the raw scores of newly tested individuals to be adjusted according to demographic variables, a correction grid was constructed, structured according to age categories, typically 10-year steps, and education level categories according to the Italian school system (see Table 5).

**TABLE 5:** Correction grid for the accuracy measures in the tasks probing aBR, NaBR and the relative control task (CTT) according to age, education and sex for the entire sample (*N* = 366).

NaBR, Frontal Body Evocation Task (FBET)												
Age	18–30		31–40		41–50		51–60		61–70		71–80	
	0		−12.35		−24.71		−37.077		−49.43		−61.79	
**Christmas Tree Task (CTT)**												
**Age**												
**Education**	**18–30**		**31–40**		**41–50**		**51–60**		**61–70**		**71–80**	
0–8	4.67		−6.93		−18.55		−30.16		−41.75		−53.38	
9–13	9.34		−2.26		−13.87		−25.48		−37.10		−48.71	
> 13	14.02		2.41		−9.20		−20.81		−32.42		−44.03	
**aBR, Hand Laterality Task (HLT)**												
**Age**												
**Education**	**18–30**		**31–40**		**41–50**		**51–60**		**61–70**		**71–80**	
	**M**	**F**	**M**	**F**	**M**	**F**	**M**	**F**	**M**	**F**	**M**	**F**
0–8	−1.13	−0.62	−0.56	0.17	−0.32	0.40	−0.09	0.63	0.13	−	0.37	1.10
9–13	−1.55	−0.82	−1.32	−0.58	−1.08	−0.35	−0.85	−0.11	−0.61	0.11	−0.38	0.35
> 13	−2.31	−1.57	−2.07	−1.34	−1.84	−1.10	−1.60	−0.87	−1.37	−0.64	−1.14	−0.40

NaBR, Nonaction-Oriented Body Representation; aBR, Action-Oriented Body Representation; M, Male; F, Female.

**FIGURE 2:**
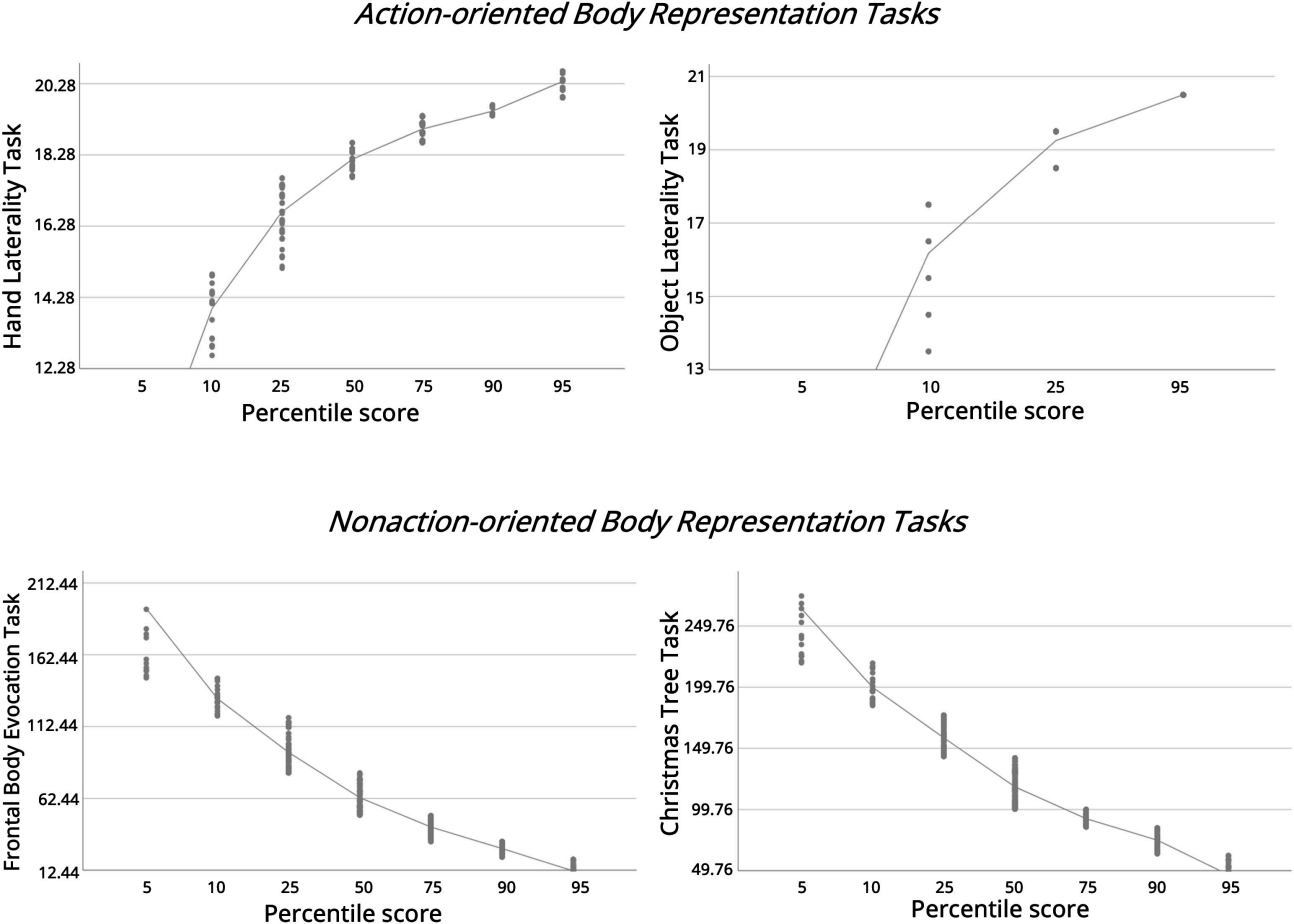
Percentile curves for adjusted scores on the body representation and control tasks. Each graph displays the relationship between percentile scores (x-axis) and task performance (y-axis) for a specific task (lab-based version): Hand Laterality Task, Object Laterality Task (control task), Frontal Body Evocation Task, and Christmas Tree Task (control task). For the Laterality tasks, higher scores indicate better accuracy (more correct responses). For the Frontal Body Evocation and Christmas Tree tasks, lower scores represent better accuracy (fewer millimeters of deviation from correct location). Note. Scores were adjusted for demographic covariates as follows: Hand Laterality Task—age, education, sex; Object Laterality Task—no adjustment; Frontal Body Evocation Task—age only; Christmas Tree Task—age and education.

Moreover, we provided a percentile distribution of adjusted scores for each body representation task and the relative control tasks (see Table 6; see Figure 2).

**TABLE 6:** The percentiles for adjusted accuracy measures for body representation and control tasks (lab-based version) for the entire sample (*N* = 366).

Percentile							
Task	5th	10th	25th	50th	75th	90th	95th
Frontal Body Evocation Task	146.16	119.85	80.12	50.74	32.38	21.46	12.44
Christmas Tree Task	219.51	184.74	142.46	109.85	85.09	63.58	49.76
Hand Laterality Task	12.28	14.93	17.65	18.63	19.39	19.89	20.00
Object Laterality Task	12.50	17.00	19.00	20.00	20.00	20.00	20.00

For the Frontal Body Evocation Task and the Christmas Tree Task performance is measured in terms of mm of deviations from the correct location — where a higher score indicates worse performance; for the Hand Laterality Task and the Object Laterality Task performance is measured in terms of the number of correct answers — where a higher score indicates better performance.

Analysis of the correlation coefficient between lab-based and web-based versions of aBR, NaBR and paired control tasks revealed a statistically significant and strong correlation (HLT, *r_*s*_* = 0.842; OLT, *r_*s*_* = 0.507; FBET, *r_*s*_* = −0.835; CTT, *r_*s*_* = −0.888; *ps* < 0.001; for the FBET and the CTT the correlations were negative since, in the laboratory-based version, performance was measured in terms of mm of deviations from the correct location—where a higher score indicates worse performance; in contrast, in the web-based version, the number of correct answers was recorded—where a higher score indicates better performance). Means and standard deviations of web-based version tasks taking into account demographical variables are reported in Supplementary Material 2.

### 3.2 Response time

Table 7 shows the descriptive statistics for aBR, NaBR, and control task response times divided by sex, age and education in the laboratory setting.

**TABLE 7:** Descriptive statistics of the total response time (in seconds) for body representation and control tasks (lab-based version) by age, education and sex for the entire sample (*N* = 366).

Education	Task							
	Age 18–30		Age 18–30		Age 18–30		Age 18–30	
	Frontal Body Evocation Task		Christmas Tree Task		Hand Laterality Task		Object Laterality Task	
	M	F	M	F	M	F	M	F
0–8	–	33.53 ± 13.52	–	47.39 ± 21.26	–	31.16 ± 5.79	–	35.51 ± 1.53
9–13	37.06 ± 13.84	35.19 ± 8.62	36.96 ± 12.48	34.16 ± 9.14	42.71 ± 22.19	31.47 ± 13.67	46.73 ± 36.19	31.43 ± 13.76
> 13	47.68 ± 32.09	31.92 ± 9.95	47.55 ± 27.19	36.97 ± 10.11	33.45 ± 15.94	26.97 ± 4.55	45.77 ± 27.51	24.93 ± 7.13
**Education**	**Age 31–40**		**Age 31–40**		**Age 31–40**		**Age 31–40**	
	**Frontal Body Evocation Task**		**Christmas Tree Task**		**Hand Laterality Task**		**Object Laterality Task**	
	**M**	**F**	**M**	**F**	**M**	**F**	**M**	**F**
0–8	45.19 ± 11.91	28.25 ± 11.48	36.02 ± 7.26	40.55 ± 29.25	53.80 ± 43.26	28.90 ± 13.25	42.18 ± 25.50	61.50 ± 57.85
9–13	35.79 ± 12.77	36.57 ± 13.87	41.82 ± 23.27	38.84 ± 14.65	31.18 ± 10.66	40.17 ± 33.69	40.44 ± 25.77	31.20 ± 28.11
> 13	35.56 ± 12.09	25.64 ± 7.06	40.50 ± 30.36	30.84 ± 10.72	32.96 ± 17.04	30.28 ± 8.87	30.15 ± 22.31	32.99 ± 16.33
**Education**	**Age 41–50**		**Age 41–50**		**Age 41–50**		**Age 41–50**	
	**Frontal Body Evocation Task**		**Christmas Tree Task**		**Hand Laterality Task**		**Object Laterality Task**	
	**M**	**F**	**M**	**F**	**M**	**F**	**M**	**F**
0–8	29.22 ± 10.94	36.33 ± 17.37	33.68 ± 14.29	40.58 ± 25.21	38.81 ± 11.31	39.24 ± 23.72	36.05 ± 9.79	52.48 ± 43.93
9–13	36.54 ± 19.94	40.69 ± 27.23	42.95 ± 24.53	46.78 ± 28.18	54.31 ± 44.44	43.14 ± 24.60	57.62 ± 40.29	34.18 ± 17.06
> 13	31.75 ± 11.18	32.92 ± 10.22	36.97 ± 6.26	38.38 ± 10.50	41.55 ± 25.59	51.30 ± 43.10	36.54 ± 19.32	34.07 ± 10.90
**Education**	**Age 51–60**		**Age 51–60**		**Age 51–60**		**Age 51–60**	
	**Frontal Body Evocation Task**		**Christmas Tree Task**		**Hand Laterality Task**		**Object Laterality Task**	
	**M**	**F**	**M**	**F**	**M**	**F**	**M**	**F**
0–8	43.26 ± 12.17	59.69 ± 25.69	41.37 ± 10.89	62.84 ± 27.19	36.94 ± 13.74	63.84 ± 51.31	39.19 ± 8.36	49.08 ± 29.44
9–13	43.89 ± 13.81	37.42 ± 18.18	45.68 ± 17.99	36.95 ± 12.91	45.62 ± 27.97	71.07 ± 50.80	47.58 ± 28.10	39 ± 29.34
> 13	44.54 ± 12.03	61.99 ± 31.94	52.83 ± 26.40	51.79 ± 19.24	74.45 ± 26.78	64.41 ± 62.56	80.86 ± 52.41	50.55 ± 46.26
**Education**	**Age 61–70**		**Age 61–70**		**Age 61–70**		**Age 61–70**	
	**Frontal Body Evocation Task**		**Christmas Tree Task**		**Hand Laterality Task**		**Object Laterality Task**	
	**M**	**F**	**M**	**F**	**M**	**F**	**M**	**F**
0–8	53.44 ± 7.28	70.36 ± 41.08	81.43 ± 3.91	77.35 ± 59.83	59.32 ± 2.84	95.01 ± 70.09	85.14 ± 37.44	79.20 ± 81.54
9–13	67.49 ± 30.28	67.92 ± 40.40	89.83 ± 31.91	82.70 ± 38.93	56.74 ± 23.42	83.15 ± 51.54	71.09 ± 47.43	79.79 ± 48.12
> 13	63.86 ± 25.89	65.55 ± 25.04	77.22 ± 37.06	71.74 ± 34.83	42.56 ± 16.67	72.51 ± 56.19	74.43 ± 40.73	63.82 ± 26.77
**Education**	**Age 71–80**		**Age 71–80**		**Age 71–80**		**Age 71–80**	
	**Frontal Body Evocation Task**		**Christmas Tree Task**		**Hand Laterality Task**		**Object Laterality Task**	
	**M**	**F**	**M**	**F**	**M**	**F**	**M**	**F**
0–8	94.66 ± 50.44	91.39 ± 49.92	107.21 ± 29.45	86.52 ± 44.41	81.08 ± 54.31	102.03 ± 51.01	83.51 ± 51.25	69.58 ± 32.51
9–13	78.08 ± 26.07	82.84 ± 11.66	88.05 ± 36.64	104.49 ± 31.94	52.90 ± 13.50	106.55 ± 77.01	49.67 ± 19.57	78.39 ± 34.09
> 13	90.29 ± 22.34	–	104.97 ± 18.68	–	116.68 ± 44.22	–	74.70 ± 19.15	–

Values are presented as mean ± standard deviation. For the number of male (M) and female (F) participants across the six age bands and the three education levels, see Table 1.

A regression model was constructed for the total response times on the HLT, which revealed that age and sex were significant in predicting the HLT response times. Age significantly influenced the OLT, FBET, and CTT response times, while education and sex did not significantly affect response times.

Overall, these results suggest that age is a significant predictor across different tasks, while sex may only affect the aBR (see Table 4).

In order to allow the response times of newly tested individuals to be adjusted according to demographic variables, a correction grid was constructed, structured according to age categories, typically 10-year steps, and education level categories according to the Italian school system (see Table 8).

**TABLE 8:** Correction grid for the total response times (in seconds) in the tasks probing aBR (HLT), NaBR (FBET) and the relative control task (OLT and CTT) according to age, and sex for the entire sample (*N* = 366).

NaBR, Frontal Body Evocation Task (FBET)												
Age	18–30		31–40		41–50		51–60		61–70		71–80	
	0		−10.24		−20.48		−30.72		−40.96		−51.21	
**Christmas Tree Task (CTT)**												
**Age**	**18–30**		**31 – 40**		**41–50**		**51–60**		**61–70**		**71–80**	
	0		−12.01		−24.02		−36.03		−48.04		−60.05	
**aBR, Hand Laterality Task (HLT)**												
**Age**	**18–30**		**31–40**		**41–50**		**51–60**		**61–70**		**71–80**	
	**M**	**F**	**M**	**F**	**M**	**F**	**M**	**F**	**M**	**F**	**M**	**F**
	10.30	0	−0.86	−10.90	−11.77	−21.81	−22.67	−32.71	−33.58	−43.62	−44.48	−54.52
**aBR, Object Laterality Task (OLT)**												
**Age**	**18–30**		**31–40**		**41–50**		**51–60**		**61–70**		**71–80**	
	0		−8.25		−16.51		−24.77		−33.03		−41.29	

NaBR, Nonaction-Oriented Body Representation; aBR, Action-Oriented Body Representation; M, Male; F, Female.

Moreover, we provided a percentile distribution of adjusted total response times for each body representation task and the relative control task (see Table 9).

**TABLE 9:** The percentiles for adjusted total response times (in seconds) of body representation and control tasks (lab-based version) for the entire sample (N = 366).

Task	Percentile						
	5th	10th	25th	50th	75th	90th	95th
Frontal Body Evocation Task	16.77	23.11	31.70	44.21	57	73.38	94.65
Christmas Tree Task	15.74	20.91	32.80	47.81	62.40	85.50	105.46
Hand Laterality Task	12.31	17.30	28.95	44.36	61.76	102.10	129.92
Object Laterality Task	9.29	12.19	20.64	32.04	51.07	89.67	115.19

Means and standard deviations of web-based version tasks taking into account demographical variables are reported in Supplementary Material 3.

## 4 Discussion

The present study is the first to provide age-, education-, and sex-stratified normative data obtained from a large sample of healthy individuals, for body representation tasks (aBR and NaBR) and paired control tasks, to disentangle the effect of cognitive functions required for performing the tasks but independent of body representation processing.

These data are particularly relevant because, while it is increasingly evident that body representation deficits are widespread after stroke (Raimo et al., 2022, 2024; Bassolino et al., 2022; Razmus, 2017; Schwoebel and Coslett, 2005), with a selective deficit present in more than one-third of patients with unilateral brain damage (i.e., 37.5%, see Raimo et al., 2022), there is, on the other hand, a clear lack of robust assessment tools to evaluate them (see Serrada et al., 2023; for a similar argument in children with motor neuron lesions see Marsico et al., 2022). In addition, these disorders may be associated with clinical variables, such as motor dysfunction and neurological disability (Butti et al., 2019). Aside from pathological conditions, it is known that mental representations of the body decline with physiological aging. Indeed, previous studies on healthy individuals (Raimo et al., 2021a; Raimo et al., 2021b,Saimpont et al., 2009; Sorrentino et al., 2021) have suggested that body representations become worse over time, following an inverted U-shaped developmental curve, and would become associated with alterations in interoceptive and sensorimotor processing (Raimo et al., 2021c; Costello and Bloesch, 2017). Thus, normative data that account for possible intervening variables, such as age, are crucial for identifying individuals with specific alterations in body representations, rather than simply attributing these changes to the general decline associated with aging.

According to previous studies (Raimo et al., 2021b; Raimo et al., 2021c; Conson et al., 2020), our results showed significant effects of age, education, and sex on aBR (i.e., the HLT performance, with older age, lower levels of education, and female sex to be associated with lower performances), but not on the paired control task (i.e., the OLT), suggesting that demographic variables specifically impact aBR. Also, in line with previous evidence (see Raimo et al., 2021c; Sorrentino et al., 2021), a significant effect of age was found on the NaBR (i.e., the FBET) and on its control task (CTT), for the latter there was also a significant effect of education, with older age and lower levels of education predicting worse performances. Moreover, age was found to significantly influence response times across all tasks, while sex specifically affected response times in the aBR task (i.e., the HLT). For this reason, it is important to adjust the raw scores obtained from individuals on the aBR and NaBR tasks and to refer to specific reference values (i.e., cut-off scores) when used in research and clinical contexts to distinguish between typical and atypical performance levels in various demographic groups.

Moreover, the inclusion of control tasks alongside body representation tasks allows for the differentiation between general cognitive deficits and those specifically related to mental body representation. By comparing performance on body representation tasks with control tasks that do not require body-related processing, it is possible to verify whether observed deficits are generalized cognitive impairments or specific to the mental representation of the body (for the relevance of using control tasks in neuropsychological studies, see also Bonato et al., 2012). Nevertheless, future investigations would benefit from the inclusion of direct and domain-specific cognitive assessments, such as measures of executive functioning, working memory, and attentional control, to more precisely delineate the contribution of general cognitive mechanisms from those uniquely associated with bodily processing.

Our findings about the high correlation between laboratory-based and web-based versions of the body representation tasks indicate a good convergent validity and support the use of these tasks, even in unsupervised settings, for the remote assessments of individuals who can have difficulty physically reaching a specific location for in-person testing. Some adaptations were introduced in the web-based versions of the tasks to ensure accessibility, standardization, and usability in unsupervised settings. Specifically, drag-and-drop responses in the FBET and CTT were replaced with binary (yes/no) judgments. This design decision was informed by prior pilot testing and the need to minimize variability in user interaction across diverse hardware setups (e.g., screen size, input device) and levels of digital literacy. Importantly, a screen calibration procedure using a standardized physical object (national health or ID card, ISO/IEC 7810 ID-1 format) was employed before task initiation to control for perceptual scaling and ensure uniform visual angles across devices. While these modifications inevitably result in procedural differences between lab and web implementations, the high inter-format correlations observed in our results support the convergent validity of the web-based tasks and justify their use in remote assessments. This finding is consistent with previous research indicating that cognitive data obtained online are broadly comparable to those obtained in the lab (e.g., Germine et al., 2012; Hilbig, 2016; Uittenhove et al., 2023).

Nonetheless, the differences between our two versions (i.e., laboratory-based and web-based) may limit full functional equivalence and should be carefully considered when interpreting performance across formats or transitioning these tools to clinical trials. Future validation efforts should aim to quantify equivalence more directly through formal measurement invariance testing and criterion-based comparisons. Another limitation of the web-based administration concerns the lack of direct supervision, which prevented real-time monitoring of compliance, attention, and adherence to instructions. Although participants received standardized written guidelines and completed the tasks independently, we cannot rule out the possibility of environmental distractions, multitasking, or even external assistance. Furthermore, no qualitative feedback was collected regarding user experience, perceived task difficulty, or technical issues. Future studies should incorporate brief post-assessment self-reports to capture these aspects more effectively and provide a deeper understanding of the context in which remote cognitive tasks are performed. In addition to the lack of supervision, another environmental limitation of our web-based testing was the inability to systematically document participants’ technical setup, including input method (e.g., mouse, trackpad), and physical environment. These factors may affect performance, especially in tasks involving visuospatial or fine motor processing. Although a calibration procedure was used to standardize stimulus size and prior research has demonstrated the general reliability of online cognitive data collection (e.g., Germine et al., 2012; Hilbig, 2016; Uittenhove et al., 2023), we acknowledge that uncontrolled variability may still influence task outcomes. Future implementations should incorporate structured self-report checklists or brief post-task questionnaires to capture these contextual variables, enhancing data quality and the interpretability of remote testing results.

Another relevant limitation concerns the demographic composition of the web-based cohort, which was skewed toward younger and more highly educated individuals. This imbalance may affect the generalizability of the findings. For this reason, we have not provided correction coefficients derived from the web-based data. Thus, test scores of web-based assessments should be interpreted with caution and cross-referenced with laboratory-based normative data or supplemented with additional clinical or functional information.

Additionally, the decision to categorize the age variable was made to facilitate the development of correction indices that are adaptable and practical for clinical use, ensuring accessibility and usability in real-world settings. However, the sensitivity and specificity for detecting clinically significant impairments have yet to be established empirically, as cut-off thresholds have not been validated in patient cohorts. Future studies should aim to anchor these values within clinical diagnostic frameworks, through criterion-based validation in patient groups with known body representation impairments.

In conclusion, this study validated computer-based tasks for assessing body representations in adults, which could be particularly useful for enhancing diagnostic accuracy and treatment efficacy in conditions affecting body representations. We provide relevant normative data and correction grids for clinical use, supported by strong convergent validity between laboratory-based and web-based versions.

## Data Availability

The procedure and the web-based version of the tasks (HLT, OLT, FBE, and CTT) are available in an open-access repository on the Open Science Framework at the following links: https://osf.io/csj63/ and https://osf.io/crq6u/. The two datasets are available in an open-access repository on the Open Science Framework at the following link: https://osf.io/a4wuv/files/osfstorage. Any further inquiries can be directed to the corresponding author.
