# Supplementary material for: The assessment of body representation in adults through computer-based tasks
Source: Front Psychol. 2025 Sep 4;16:1610265. doi: 10.3389/fpsyg.2025.1610265 (PMC12444331; doi:10.3389/fpsyg.2025.1610265)
Supplement: Supplementary file 1 [file Table_1.docx]

**Supplementary Material 1**. Demographic distribution of the sample underwent body representation and control tasks in the web-based version.

|  | Age (years) | | | | | | | |  | |
| --- | --- | --- | --- | --- | --- | --- | --- | --- | --- | --- |
|  | 18-30 | | 31-40 | | 41-50 | | 51-60 | | Total |  |
| Education (years) | M | F | M | F | M | F | M | F |  |  |
| 0-8 | - | - | - | - | - | - | - | 4 | 4 |  |
| 9-13 | 39 | 65 | 5 | 7 | 4 | 5 | 13 | 12 | 150 |  |
| >13 | 19 | 76 | 11 | 8 | 6 | 7 | 5 | 19 | 151 |  |
| Total | 58 | 141 | 16 | 15 | 10 | 12 | 18 | 35 | 305 |  |

M: Male; F: Female.

**Supplementary Material 2.** Descriptive statistics of the accuracy measures for body representation and control tasks (*web-based version*) by age, education, and sex for the entire sample (N = 305).

| **Education** | **Age 18-30** | | **Age 18-30** | | **Age 18-30** | | **Age 18-30** | |
| --- | --- | --- | --- | --- | --- | --- | --- | --- |
|  | Frontal Body Evocation Task | | Christmas Tree Task | | Hand Laterality Task | | Object Laterality Task | |
|  | M | F | M | F | M | F | M | F |
| 0-8 | - | - | - | - | - | - | - | - |
| 9-13 | 36.49±6.01 | 37.20±4.85 | 43.03±3.93 | 42.69±3.81 | 46.46±1.89 | 46.06±4.53 | 46.23±4.49 | 43.68±8.10 |
| >13 | 37.58±6.11 | 35.70±5.76 | 42.42±4.85 | 41.99±4.78 | 47.16±0.83 | 45.18±6.01 | 46.37±5.47 | 45.76±5.03 |
|  | | | | | | | | |
| **Education** | **Age 31-40** | | **Age 31-40** | | **Age 31-40** | | **Age 31-40** | |
|  | Frontal Body Evocation Task | | Christmas Tree Task | | Hand Laterality Task | | Object Laterality Task | |
|  | M | F | M | F | M | F | M | F |
| 0-8 | - | - | - | - | - | - | - | - |
| 9-13 | 33.20±10.03 | 34.14±7.42 | 33.20±10.91 | 36.14±10.69 | 42.60±10.43 | 39.86±10.76 | 38.60±10.62 | 40.86±10.69 |
| >13 | 36.01±5.44 | 36.63±4.71 | 41.64±7.15 | 44.38±2.38 | 43.18±8.80 | 46.25±2.49 | 43.27±7.35 | 47.25±1.38 |
|  | | | | | | | | |
| **Education** | **Age 41-50** | | **Age 41-50** | | **Age 41-50** | | **Age 41-50** | |
|  | Frontal Body Evocation Task | | Christmas Tree Task | | Hand Laterality Task | | Object Laterality Task | |
|  | M | F | M | F | M | F | M | F |
| 0-8 | - | - | - | - | - | - | - | - |
| 9-13 | 36.75±2.06 | 35.01±4.63 | 44.50±3.10 | 41.60±3.78 | 45.50±1.29 | 46.20±2.95 | 47.75±0.50 | 45.20±2.38 |
| >13 | 30.50±11.04 | 35.71±4.88 | 36.17±11.73 | 41.14±8.39 | 39.83±9.23 | 47.01±1.29 | 42.33±9.97 | 46.29±2.43 |
|  | | | | | | | | |
| **Education** | **Age 51-60** | | **Age 51-60** | | **Age 51-60** | | **Age 51-60** | |
|  | Frontal Body Evocation Task | | Christmas Tree Task | | Hand Laterality Task | | Object Laterality Task | |
|  | M | F | M | F | M | F | M | F |
| 0-8 | - | 35.75±6.39 | - | 41.50±5.01 | - | 39.25±12.23 | - | 39.25±12.23 |
| 9-13 | 36.85±7.14 | 33.58±8.50 | 44.08±4.81 | 39.58±7.10 | 43.08±8.56 | 41.25±9.88 | 42.31±10.21 | 41.42±8.73 |
| >13 | 34.20±5.54 | 35.47±6.34 | 37.60±8.62 | 42.01±4.39 | 38.80±12.55 | 42.79±7.51 | 38.80±9.62 | 44.01±6.96 |

Values are presented as mean ± standard deviation. Performance in all tasks is measured by the number of correct responses, with higher values indicating

better performance. For the number of male (M) and female (F) participants across the four age bands and the three education levels, see Supplementary Material 1.

**Supplementary Material 3**. Descriptive statistics of the total response times (in seconds) for body representation and control tasks (*web-based version*) by age, education, and sex for the entire sample (N = 305).

| **Education** | **Age 18-30** | | **Age 18-30** | | **Age 18-30** | | **Age 18-30** | |
| --- | --- | --- | --- | --- | --- | --- | --- | --- |
|  | Frontal Body Evocation Task | | Christmas Tree Task | | Hand Laterality Task | | Object Laterality Task | |
|  | M | F | M | F | M | F | M | F |
| 0-8 | - | - | - | - | - | - | - | - |
| 9-13 | 97.84±43.70 | 94.82±28.66 | 83.69±38.92 | 88.82±37.61 | 131.26±59.2 | 126.28±51.35 | 128.27±82.21 | 135.42±72.6 |
| >13 | 100.63±34.72 | 128.36±148.8 | 85.69±32.20 | 125.04±268.5 | 127.96±51.1 | 149.53±75.96 | 119.71±68.33 | 137.82±86.06 |
|  | | | | | | | | |
| **Education** | **Age 31-40** | | **Age 31-40** | | **Age 31-40** | | **Age 31-40** | |
|  | Frontal Body Evocation Task | | Christmas Tree Task | | Hand Laterality Task | | Object Laterality Task | |
|  | M | F | M | F | M | F | M | F |
| 0-8 | - | - | - | - | - | - | - | - |
| 9-13 | 95.72±40.20 | 149.10±58.50 | 73.65±51.24 | 99.96±28.96 | 122.15±42.5 | 217.95±118.2 | 91.11±38.89 | 207.78±127.6 |
| >13 | 138.53±140.2 | 118.45±109.1 | 102.38±70.58 | 83.15±24.77 | 142.24±68.4 | 111.8±32.79 | 140.81±68.86 | 86.07±17.99 |
|  | | | | | | | | |
| **Education** | **Age 41-50** | | **Age 41-50** | | **Age 41-50** | | **Age 41-50** | |
|  | Frontal Body Evocation Task | | Christmas Tree Task | | Hand Laterality Task | | Object Laterality Task | |
|  | M | F | M | F | M | F | M | F |
| 0-8 | - | - | - | - | - | - | - | - |
| 9-13 | 169.79±90.77 | 139.85±43.31 | 147.50±91.69 | 119.54±40.11 | 149.82±51.6 | 173.52±62.97 | 107.72±20.01 | 175.12±72.01 |
| >13 | 93.72±64.17 | 110.71±35.24 | 123.61±65.16 | 140.94±120.1 | 120.63±44.5 | 146.89±75.99 | 114.11±35.94 | 209.01±177.4 |
|  | | | | | | | | |
| **Education** | **Age 51-60** | | **Age 51-60** | | **Age 51-60** | | **Age 51-60** | |
|  | Frontal Body Evocation Task | | Christmas Tree Task | | Hand Laterality Task | | Object Laterality Task | |
|  | M | F | M | F | M | F | M | F |
| 0-8 | - | 196.34±149.2 | - | 213.32±211.9 | - | 1228.5±1846 | - | 445.32±386.6 |
| 9-13 | 126.10±74.08 | 150.48±60.95 | 98.98±34.86 | 141.78±47.51 | 175.31±107 | 241.2±124.41 | 157.47±192.3 | 197.39±143.4 |
| >13 | 109.86±42.06 | 138.86±45.55 | 161.91±145.8 | 127.48±35.69 | 114.5±31.1 | 192.43±119.7 | 104.09±44.37 | 198.41±118.4 |

Values are presented as mean ± standard deviation. For the number of male (M) and female (F) participants across the four age bands and the three education levels, see Supplementary Material 1.
